# D-Dimer Combined with Fibrinogen Predicts the Risk of Venous Thrombosis in Fracture Patients

**DOI:** 10.1155/2020/1930405

**Published:** 2020-09-23

**Authors:** Chaohui Lin, Yifan Chen, Bin Chen, Ke Zheng, Xiongbiao Luo, Fengfei Lin

**Affiliations:** ^1^Department of Orthopedic Surgery, Fuzhou Second Hospital Affiliated to Xiamen University, The Teaching Hospital of Fujian Medical University, Fuzhou, Fujian, China; ^2^Fujian University of Traditional Chinese Medicine, Fuzhou, Fujian, China; ^3^Xiamen University, Xiamen, Fujian, China

## Abstract

**Objective:**

While D-dimer can successfully diagnose venous thrombosis due to its excellent negative predictive value (NPV), it cannot be used to detect venous thromboembolism (VTE) because of its low positive predictive value (PPV). This study aims to investigate if a combination of using D-dimer and fibrinogen can improve PPV in the VTE diagnosis.

**Methods:**

We retrospectively analyzed various data including D-dimer, fibrinogen, C-reactive protein, ultrasound, and others collected from 10775 traumatic fracture patients and categorized them into two groups of VTE and non-VTE. By comparing the difference between the two groups, we employ multiple logistic regression to find risk factors that are useful to detect VTE. The receiver operating characteristic (ROC) curve was used to evaluate the diagnostic yield of using fibrinogen, D-dimer, and their combination, respectively. Also, these data were classified into quartiles by patient age. We perform the same analysis on the quartiles and find if the patient's age has an impact on diagnosing VTE.

**Results:**

The univariate analysis demonstrated that five factors of age, D-dimer, fibrinogen, C-reactive protein, and high-density lipoprotein cholesterol were significant to predict VTE. ROC showed that D-dimer was more useful than fibrinogen for the diagnosis of VTE, while the area under the curve (AUC) was 0.7296 for D-dimer and 0.5209 for fibrinogen. The cutoff point of D-dimer and fibrinogen was 424.89 ng/ml and 3.543 g/L, respectively. The specificity of fibrinogen was 0.777 which was better than D-dimer, while the sensitivity of fibrinogen was lower than that of D-dimer. Both PPV and NPV were similar in D-dimer and fibrinogen. The PPV of combining D-dimer and fibrinogen in ages Q3 (60 < age ≤ 70) and Q4 (age > 70) was better than using either D-dimer or fibrinogen.

**Conclusions:**

Fibrinogen is a promising strategy for the diagnosis of subclinical VTE and postoperative VTE. In particular, a combination of D-dimer and fibrinogen can improve the PPV to successfully diagnose VTE in traumatic fracture patients who are more than 60 years old. *Levels of Evidence*. This assay is a diagnostic test at level II.

## 1. Introduction

Venous thromboembolism (VTE) which includes deep venous thrombosis (DVT) and pulmonary embolism (PE) is a critical complication of fracture patients. Untreated PE leads to a mortality rate of 30% including 5–10% inpatient deaths in US [1]. An overall 1.47% symptomatic VTE rate occurred in a large amount of low-energy community-acquired isolated fractures, while there were 1.25%, 2.6%, and 0.7% VTE rates in isolated upper extremity fractures, hip fractures, and pelvis fractures, respectively [2].

Plasma D-dimer (fibrin-degradation products) measurement provides surgeons with information about fibrin formation including fibrinolysis. It is used to exclude the diagnosis of venous thrombosis (VTE) because of its excellent negative predictive value (NPV). A standard cutoff of 500 ng/ml is used in clinical practice, with values above that level considered positive. However, only an elevated D-dimer concentration cannot confirm DVT diagnosis and cannot be used to improve its positive predictive value (PPV), since increased D-dimer levels can also be detected in patients with fracture, malignancy, trauma, recent surgery, and infection. It has been reported that low soluble fibrinogen concentrations were detected in normal plasma, and high concentrations were found in patients with thrombotic disease [3]. In this research, we want to study whether soluble fibrinogen can help diagnose VTE and whether a combination of using D-dimer and fibrinogen can improve PPV in detecting VTE.

This study aims to investigate the risk factor associated with DVT and evaluate the potential of a new combination of fibrinogen and D-dimer to precisely diagnose fracture patients with VTE.

## 2. Material and Methods

### 2.1. Patients and Methods

A total of 10775 traumatic fracture patients (5500 males and 5275 females; mean age: 56.33 ± 18.70) were retrospectively selected from December 2010 to April 2017. Inclusion criteria were traumatic fracture patients who were examined by a whole-leg compression ultrasound detect DVT before and after surgery in medical records. For the patient who was diagnosed with DVT on US-contrast CT, pulmonary ventilation and perfusion were performed on him or her to search for PE. Simultaneously, we recorded all information and data including D-dimer, fibrinogen, international prothrombin ratio, low-density lipoprotein cholesterol, partial thromboplastin time, total cholesterol, blood glucose, C-reactive protein, prothrombin time, triglyceride, thrombin time, platelet count, and high-density lipoprotein cholesterol during the time of hospital admission after traumatic fracture. Exclusion criteria contain (1) history of VTE, (2) use of anticoagulant or heparin therapy before administration, (3) active cancer, and (4) pregnancy.

The patients were categorized into VTE positive and negative. We also divided the patients into four categories based on age: Q1 (age ≤ 45), Q2 (45 < age ≤ 60), Q3 ((60 < age ≤ 70), and Q4 (age > 70).

As our study is a retrospective study, ethical approval was not necessary.

### 2.2. Statistical Analysis

Since the use of fibrinogen and D-dimer for identifying fracture patients with VTE was not well documented, there is no standard to select a sample size. A convenient sample size is usually set to around 1000 patients, which was generally agreed on by the steering committee.

We employed Student's test to find the difference between the continuous clinical characters of patients in the VTE and non-VTE groups. Chi-square tests were performed to analyze the categorical characters in two groups. Potential risk factors that were used to predict VTE were then selected through a multiple logistic regression with backward stepwise selection. The fit and predictive accuracy of the final model were evaluated by the Hosmer–Lemeshow test. The receiver operating characteristic (ROC) curve was used to assess the diagnostic yield of using fibrinogen, D-dimer, and their combination. The cutoff points of these two indicators were selected according to the maximum Youden index. Sensitivity, specificity, NPV, and PPV and their 95% confidence intervals (CI) for VTE diagnosis were also measured. A comparison of the diagnostic ability of these two indicators was performed via area under the ROC curve with nonparametric approach of DeLong et al. [4]. To test age influence, the patients were divided into quartiles in light of their age. And the above analyses were also performed according to age groups. All analyses were carried out with SAS version 9.4 (SAS Institute Inc, Cary, NC, USA). Statistical significance was considered as 95% confidence interval (CI) that did not include zero or *p* < 0.05 (two-sided).

## 3. Results

Of the total, 10775 patients were included in the present study (flow diagram). The validation results showed the significant differences in the international prothrombin ratio, thrombin time, prothrombin time, total cholesterol, triglyceride, C-reactive protein, D-dimer, fibrinogen, low-density lipoprotein cholesterol, blood glucose, and age values between VTE-negative and VTE-positive patients. However, there was no significant difference in partial thromboplastin time and high-density lipoprotein cholesterol between VTE-negative and VTE-positive patients (Table 1).

The results of the univariate and multiple logistic regression models are shown in Table 2. In the univariate analysis, we found that the five factors of age, D-dimer, fibrinogen, C-reactive protein, and high-density lipoprotein cholesterol play a significant role in detecting VTE.

**Figure 1:**
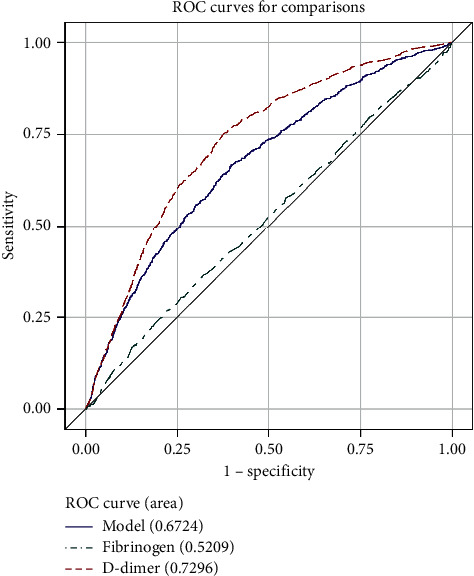
ROC curve for comparisons of D-dimer, fibrinogen, and combination of them in all patients.

**Figure 2:**
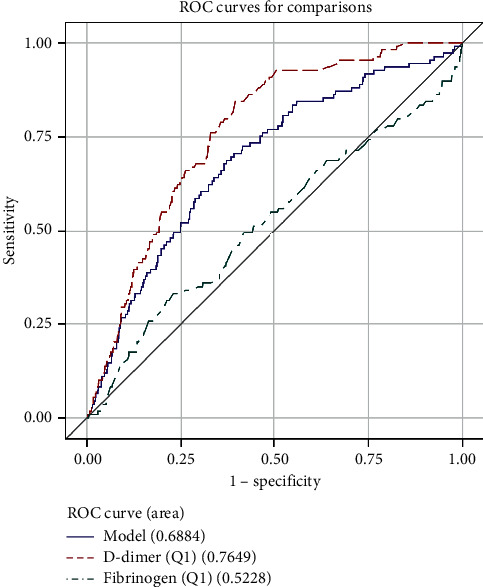
ROC curve for comparisons of D-dimer, fibrinogen, and combination of them in patients (age ≤ 45).

**Figure 3:**
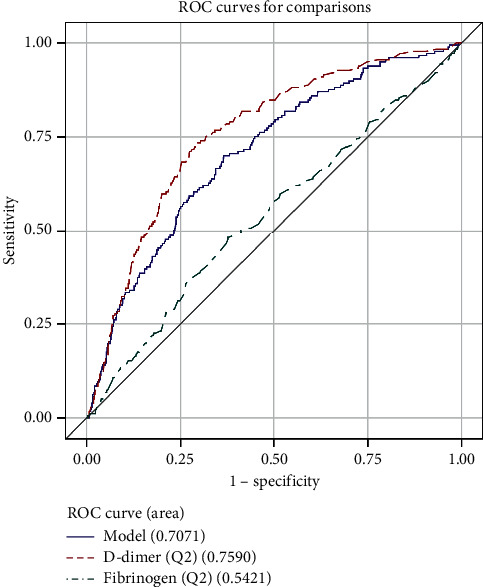
ROC curve for comparisons of D-dimer, fibrinogen, and combination of them in patients (45 < age ≤60).

**Figure 4:**
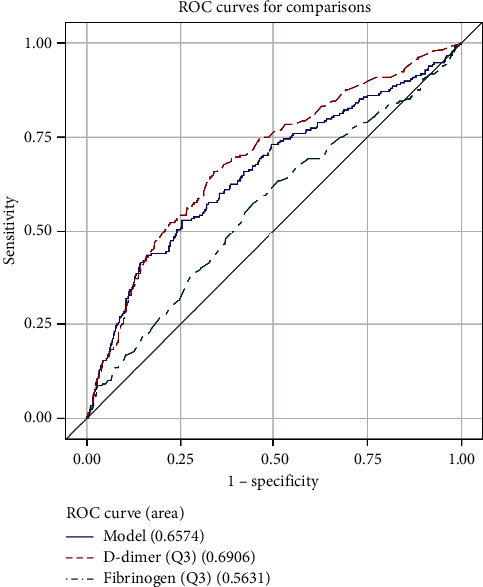
ROC curve for comparisons of D-dimer, fibrinogen, and combination of them in patients (60 < age ≤ 70).

**Figure 5:**
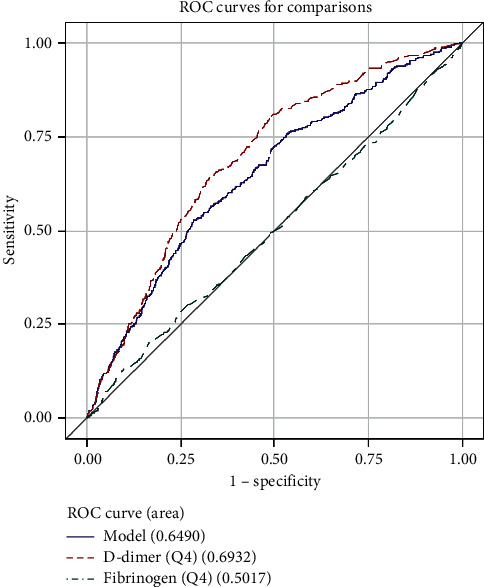
ROC curve for comparisons of D-dimer, fibrinogen, and combination of them in patients (age > 70).

The ROC analysis shows that D-dimer was more useful than fibrinogen for the diagnosis of VTE. The area under the curve (AUC) was 0.7296 in D-dimer and 0.5209 in fibrinogen (Figure 1). The AUC of the combination of D-dimer and fibrinogen was 0.6724. The cutoff point was 424.89 ng/ml and 3.543 g/L for D-dimer and fibrinogen, respectively. The AUC of different ages is shown in Table 3 and Figures 2–5.

The sensitivity, specificity, PPV, NPV, and their respective 95% confidence intervals were calculated for D-dimer, fibrinogen, and their combination in Table 4. The specificity of fibrinogen is 0.777 which was better than D-dimer, but the sensitivity of fibrinogen was lower than that of D-dimer. The PPV and NPV were similar in D-dimer and fibrinogen. The PPV of D-dimer and fibrinogen combination in Q3 and Q4 was better than solely using D-dimer or fibrinogen.

## 4. Discussion

It is well known that D-dimer has poor specificity for the diagnosis of DVT and/or PE. Extravascular fibrin degrades into D-dimer by local fibrinolytic enzymes. D-dimer can be easily diffused into the bloodstream due to their low molecular weight. This conception can be supported by elevated D-dimer levels that are found in cancer or acute inflammatory patients [5, 6]. Previous studies found that while D-dimer has a high negative predictive value for excluding DVT and PE, the positive predictive value for DVT and/or PE is very poor. Therefore, D-dimer is only valuable as an exclusionary test [7, 8]. On the other hand, plasma fibrinogen cannot come from inflammatory or neoplastic sites for its high molecular weight. So, the presence of fibrinogen in plasma is a determination for the activation of intravascular coagulation [9]. A multiethnic cohort observed that smokers have higher fibrinogen levels than nonsmokers, which implies that smoke exposures cause hyperfibrinogenemia which increases thrombosis in cardiovascular patients [10]. While the guidelines from the American College of Chest Physicians in 2012 recommend using a score for the assessment of risk factor for VTE such as the Padua Prediction Score (PPS) [11], the research from Italy suggests that adding the clot waveform analysis, fibrinogen, and D-dimer into PPS provides better identification of patients with a VTE risk [3]. Our study indicated that D-dimer and fibrinogen were significantly different between VTE-negative and VTE-positive patients. In the univariate analysis, a combination of D-dimer and fibrinogen meets the criteria for the VTE prediction.

We also found significant differences in the prothrombin international ratio, partial thromboplastin time, thrombin time, prothrombin time, total cholesterol, triglyceride, C-reactive protein, blood platelet, low-density lipoprotein cholesterol, blood glucose, and age values besides D-dimer and fibrinogen between VTE-negative and VTE-positive patients. In the univariate analysis, we found that the five factors of age, D-dimer, fibrinogen, C-reactive protein, and high-density lipoprotein cholesterol are important to predict VTE. This result was similar to the increase in serum lipoprotein and fibrinogen levels in Chinese women during pregnancy and puerperium [12].

The AUC area was larger in young patients (age ≤ 60) than in old patients (age > 60) in D-dimer. It is similar to many works where the age-adjusted strategy for D-dimer interpretation used a progressively higher cutoff to categorize results as positive in old patients [13]. This age-adjusted threshold increases the specificity of D-dimer testing compared to use the conventional cutoff value in patients in all ages [14]. This age-adjusted cutoff value might be applied to patients older than 50 years, which can improve the specificity for VTE [15]. We also believe that the AUC area of fibrinogen was larger in patients (age ≤ 70) than patients older than 70. It suggests that the age-adjusted strategy for fibrinogen is also needed.

As the AUCs of D-dimer and fibrinogen were not low in the ROC analysis, we believe that they are useful for the diagnosis of VTE. In particular, D-dimer was more useful than fibrinogen for the diagnosis of VTE in all ages as the former's AUC was larger than that of the latter. The cutoff value of D-dimer (424.89 ng/ml) was similar to 500 ng/ml in Europe and North America [16].

When the endothelium is injured in the fracture, the subendothelial matrix and fibrinogen thrombospondin were exposed, and the subendothelial matrix proteins bind to glycoprotein Ib on the platelet surface via vWF and initiate primary hemostasis. Platelets also release fibrinogen from intracellular stores. Fracture is accompanied by increasing concentrations of factor VII and X through the extrinsic pathway of hemostasis and pronouncing increases in fibrinogen [17]. It indicates that fibrinogen is increasing early during coagulation. Our research found that the fibrinogen assays were potentially useful for the prediction of postoperative VTE, which is considered to be pre-VTE before surgery after fracture. The AUC and odds ratio for predicting VTE were lower in fibrinogen than in D-dimer. But the sensitivity of fibrinogen was 57.7% in age Q3 which was better than other ages, indicating that fibrinogen was more useful for patients aged 60 to 75. These findings showed that increased fibrinogen levels indicate a hypercoagulable state and an increased risk of thrombosis. It might be useful to suggest that these patients receive anticoagulants before and after surgery. Anticoagulants like fondaparinux and rivaroxaban were approved as prophylaxis drugs for orthopedic surgery, but these sometimes cause severe bleeding [18]. The current results showed that anticoagulants might be recommended in patients with fibrinogen and D-dimer higher than 3.543 g/L and 424.89 ng/ml, respectively. It is therefore considered that we can administer anticoagulant treatments for patients for prevention of VTE after fracture and orthopedic surgery according to fibrinogen and D-dimer levels. Meanwhile, we can let patients do whole-leg compression ultrasound, pulmonary ventilation and perfusion, computed tomography pulmonary arteriography, and echocardiography to exclude thromboembolism when fibrinogen and D-dimer are higher than 3.543 g/L and 424.89 ng/ml, respectively. And no such checks are required if just D-dimer was higher than 3.543 g/L.

Also, fracture reduced movement and increased bleeding, blood viscosity, and concurrent infection. These factors can result in the hypercoagulable state and fibrinogen increase. In our study, the specificity of fibrinogen was better than that of D-dimer, while the sensitivity of fibrinogen was lower than that of D-dimer. Because of the heterogeneity of the causes of a raised of D-dimer and fibrinogen, these tests could not be used for its PPV [19]. In agreement with this, we have a low PPV in our findings (Table 4). But we found that PPV combined with D-dimer and fibrinogen was better than their PPV alone in age Q3 and age Q4. It indicates that fibrinogen combined with D-dimer was useful to diagnose VTE in patients older than 60 years.

## 5. Limitations

First, our work is a retrospective study. For D-dimer and fibrinogen measured at the time of hospital admission after traumatic fracture, we cannot determine whether D-dimer and fibrinogen can be useful for diagnosis of VTE in patients on days 1, 7, and 14 after orthopedic surgery. It requires further studies to verify the guess. Second, we only analyzed patients with fractures, which limits broader applications. We will include cancer, infection diseases, disseminated intravascular coagulation, replacement arthroplasty, diabetes mellitus, and heart disease paients for analysis in the future. Finally, in our study, we used an age-adjusted interpretation, and perhaps we will use a clinical probability-adjusted D-dimer and fibrinogen in future research.

## 6. Conclusion

Our investigation suggests that fibrinogen is a promising component for the diagnosis of subclinical VTE and postoperative VTE. In particular, for patients more than 60 years old, the combination of using D-dimer and fibrinogen can improve the PPV to diagnose VTE in fracture patients.

## Figures and Tables

**Table 1:** Clinical characteristics of patients in VTE and non-VTE groups.

Variables	Non-VTE	VTE	*p* value
Prothrombin international ratio	0.98 ± 0.12	1.01 ± 0.15	<0.0001
Partial thromboplastin time	29.50 ± 5.65	29.33 ± 5.80	0.4269
Thrombin time	19.42 ± 2.81	18.95 ± 2.52	<0.0001
Prothrombin time	11.63 ± 1.45	11.85 ± 1.81	0.0007
Total cholesterol	4.76 ± 1.17	4.57 ± 1.17	<0.0001
Triglyceride	1.46 ± 1.14	1.31 ± 0.97	<0.0001
C-reactive protein	21.93 ± 40.26	34.94 ± 46.39	<0.0001
D-dimer	1079.84 ± 2769.37	2505.34 ± 4086.32	<0.0001
Fibrinogen	3.04 ± 0.95	3.11 ± 0.98	0.0458
Blood platelet	244.84 ± 80.19	225.42 ± 80.21	<0.0001
Low-density lipoprotein cholesterol	2.59 ± 0.84	2.46 ± 0.83	<0.0001
High-density lipoprotein cholesterol	1.43 ± 0.43	1.42 ± 0.41	0.3346
Blood glucose	5.75 ± 1.85	6.18 ± 2.30	<0.0001
Age	55.68 ± 18.72	64.08 ± 16.65	<0.0001

VTE: venous thromboembolism

**Table 2:** Results of univariate and multiple logistic regression models of the predictive risk factor of VTE in fracture patients.

Variables	Venous thrombosis	
	Univariate model^a^ OR (95%CI)	Adjusted model^b^ OR (95%CI)
Age		
Q1	Reference	Reference
Q2	1.61 (1.26, 2.05)	2.16 (1.21, 3.90)
Q3	2.01 (1.58, 2.55)	2.82 (1.59, 5.03)
Q4	3.40 (2.72, 4.23)	4.34 (2.46, 7.68)
D-dimer		
M	Reference	Reference
H	4.78 (3.75, 6.08)	2.55 (1.79, 3.64)
Fibrinogen		
L	Reference	Reference
M	1.08 (0.79, 1.50)	0.36 (0.16, 0.80)
H	0.80 (0.61, 1.05)	0.73 (0.39, 1.36)
C-reactive protein		
M	Reference	Reference
H	2.56 (2.20, 2.96)	1.59 (1.14, 2.36)
High-density lipoprotein cholesterol		
L	Reference	Reference
M	0.97 (0.50, 1.87)	0.22 (0.08, 0.62)
H	1.06 (0.56, 2.03)	0.21 (0.08, 0.59)

^a^Without adjusting for other covariates; ^b^adjusted for other covariates in multiple regression with backward selection; Q1 (age ≤ 45), Q2 (45 < age ≤ 60), Q3 ((60 < age ≤ 70), and Q4 (age > 70).

**Table 3:** The cutoff point and AUC of different ages.

ROC model	AUC				Cutoff point
	Area	Standard error	95% Wald confidence limits		
D-dimer	0.7296	0.0086	0.7128	0.7464	424.89
D2-AgeQ1	0.7649	0.0196	0.7265	0.8032	
D2-AgeQ2	0.7590	0.0181	0.7235	0.7944	
D2-AgeQ3	0.6906	0.0197	0.6520	0.7293	
D2-AgeQ4	0.6932	0.0141	0.6655	0.7208	
Fibrinogen	0.5209	0.0106	0.5002	0.5417	3.543
Fib-AgeQ1	0.5228	0.0311	0.4618	0.5837	
Fib-AgeQ2	0.5421	0.0232	0.4966	0.5876	
Fib-AgeQ3	0.5631	0.0216	0.5208	0.6054	
Fib-AgeQ4	0.5017	0.0169	0.4685	0.5349	

AUC: area under the curve; D2: D-dimer; Fib: fibrinogen; Q1 (age ≤ 45), Q2 (45 < age ≤ 60), Q3 ((60 < age ≤ 70), and Q4 (age > 70);

**Table 4:** Sensitivity, specificity, positive predictive value, and negative predictive value of D-dimer, fibrinogen, and combination of D-dimer and fibrinogen based on the Youden index.

Variable	Value	Se	Sp	PPV	NPV	Accuracy
D2	0.375	0.752 (0.722, 0.781)	0.624 (0.615, 0.633)	0.144 (0.133, 0.154)	0.968 (0.963, 0.972)	0.634 (0.625, 0.643)
Fib	0.046	0.269 (0.239, 0.298)	0.777 (0.769, 0.785)	0.092 (0.081, 0.103)	0.927 (0.921, 0.932)	0.738 (0.730, 0.746)
D2 + FIB	0.271	0.668 (0.636, 0.700)	0.603 (0.594, 0.613)	0.124 (0.114, 0.133)	0.956 (0.951, 0.961)	0.608 (0.599, 0.618)
Q1						
D2	0.450	0.844 (0.776, 0.912)	0.606 (0.588, 0.625)	0.083 (0.067, 0.099)	0.989 (0.984, 0.994)	0.616 (0.598, 0.634)
Fib	0.101	0.330 (0.242, 0.419)	0.771 (0.755, 0.787)	0.057 (0.039, 0.076)	0.965 (0.957, 0.973)	0.753 (0.737, 0.770)
D2 + FIB	0.318	0.688 (0.601, 0.775)	0.630 (0.611, 0.648)	0.073 (0.057, 0.089)	0.980 (0.973, 0.986)	0.632 (0.614, 0.650)
Q2						
D2	0.445	0.735 (0.669, 0.800)	0.710 (0.693, 0.728)	0.147 (0.123, 0.170)	0.975 (0.968, 0.982)	0.712 (0.695, 0.729)
Fib	0.104	0.480 (0.407, 0.554)	0.624 (0.605, 0.642)	0.080 (0.063, 0.096)	0.947 (0.936, 0.957)	0.615 (0.597, 0.633)
D2 + FIB	0.336	0.701 (0.633, 0.768)	0.635 (0.616, 0.654)	0.115 (0.096, 0.134)	0.969 (0.961, 0.977)	0.639 (0.622, 0.657)
Q3						
D2	0.321	0.659 (0.594, 0.723)	0.662 (0.643, 0.681)	0.142 (0.120, 0.164)	0.958 (0.949, 0.968)	0.662 (0.644, 0.680)
Fib	0.133	0.577 (0.510, 0.644)	0.556 (0.536, 0.575)	0.099 (0.082, 0.116)	0.939 (0.927, 0.952)	0.557 (0.538, 0.576)
D2 + FIB	0.275	0.529 (0.461, 0.597)	0.746 (0.729, 0.763)	0.150 (0.124, 0.176)	0.949 (0.939, 0.959)	0.729 (0.712, 0.746)
Q4						
D2	0.318	0.806 (0.765, 0.847)	0.512 (0.492, 0.532)	0.191 (0.171, 0.211)	0.948 (0.937, 0.960)	0.549 (0.530, 0.567)
Fib	0.036	0.200 (0.158, 0.242)	0.836 (0.821, 0.850)	0.149 (0.117, 0.180)	0.880 (0.866, 0.893)	0.756 (0.740, 0.772)
D2 + FIB	0.247	0.530 (0.478, 0.582)	0.718 (0.700, 0.735)	0.212 (0.185, 0.239)	0.914 (0.902, 0.927)	0.694 (0.677, 0.711)

D2: D-dimer; FIB: fibrinogen; Se: sensitivity, Sp: specificity; PPV: positive predictive value; NPV: negative predictive value; Q1 (age ≤ 45), Q2 (45 < age ≤ 60), Q3 ((60 < age ≤ 70), and Q4 (age > 70);

## Data Availability

The data used to support the findings of the study are included in the supplementary information files.

## Supplementary Materials

Supplementary Materials Data of all included patients.
